# CD64-Neutrophil expression and stress metabolic patterns in early sepsis and severe traumatic brain injury in children

**DOI:** 10.1186/1471-2431-13-31

**Published:** 2013-03-01

**Authors:** Diana-Michaela Fitrolaki, Helen Dimitriou, Maria Kalmanti, George Briassoulis

**Affiliations:** 1 Pediatric Intensive Care Unit, University Hospital of Heraklion, 71110 Heraklion, Crete, Greece; 2 Department of Pediatric Hematology-Oncology, University of Crete, Medical School, Crete, Greece

**Keywords:** Metabolic pattern, Glucose, Lipoproteins, CD64, CD11b, Neutrophils, Sepsis, Traumatic brain injury

## Abstract

**Background:**

Critical illness constitutes a serious derangement of metabolism. The aim of our study was to compare acute phase metabolic patterns in children with sepsis (S) or severe sepsis/septic shock (SS) to those with severe traumatic brain injury (TBI) and healthy controls (C) and to evaluate their relations to neutrophil, lymphocyte and monocyte expressions of CD64 and CD11b.

**Methods:**

Sixty children were enrolled in the study. Forty-five children with systemic inflammatory response syndrome (SIRS) were classified into three groups: TBI (n = 15), S (n = 15), and SS (n = 15). C consisted of 15 non- SIRS patients undergoing screening tests for minor elective surgery. Blood samples were collected within 6 hours after admission for flow cytometry of neutrophil, lymphocyte and monocyte expression of CD64 and CD11b (n = 60). Procalcitonin (PCT), C-reactive protein (CRP), glucose, triglycerides (TG), total cholesterol (TC), high (HDL) or low-density-lipoproteins (LDL) were also determined in all groups, and repeated on day 2 and 3 in the 3 SIRS groups (n = 150).

**Results:**

CRP, PCT and TG (p < 0.01) were significantly increased in S and SS compared to TBI and C; glucose did not differ among critically ill groups. Significantly lower were the levels of TC, LDL, and HDL in septic groups compared to C and to moderate changes in TBI (p < 0.0001) but only LDL differed between S and SS (p < 0.02). Among septic patients, PCT levels declined significantly (p < 0.02) with time, followed by parallel decrease of HDL (p < 0.03) and increase of TG (p < 0.02) in the SS group. Neutrophil CD64 (nCD64) expression was higher in patients with SS (81.2%) and S (78.8%) as compared to those with TBI (5.5%) or C (0.9%, p < 0.0001). nCD64 was positively related with CRP, PCT, glucose, and TG (p < 0.01) and negatively with TC, LDL, and HDL (p < 0.0001), but not with severity of illness, hematologic indices, length of stay or mechanical ventilation duration.

**Conclusions:**

In sepsis, the early stress-metabolic pattern is characterized by a high (nCD64, glucose, TG) - low (TC, HDL, LDL) combination in contrast to the moderate pattern of TBI in which only glucose increases combined with a moderate cholesterol - lipoprotein decrease. These early metabolic patterns persist the first 3 days of acute illness and are associated with the acute phase CD64 expression on neutrophils.

## Background

Sepsis may be elicited through either pathogen-associated or danger-associated (in the absence of infection) molecular patterns (PAMPs or DAMPs, respectively) as a systemic inflammatory response syndrome (SIRS) [1]. Once primed by bacterial products and endogenous mediators, neutrophils are critical effector cells during acute inflammatory processes [2]. The CD64 is a membrane glycoprotein that mediates endocytosis, phagocytosis, antibody-dependent cellular toxicity, cytokine release, and superoxide generation. It is constitutively expressed on monocytes and macrophages. It is expressed at low concentration on the surface of non-activated neutrophils, but can be markedly upregulated at the onset of sepsis [3]. Elevated monocyte CD64 expression was reported in adult patients with sepsis [4] and in a mixed group of neonates and children with SIRS and sepsis [5]. Neutrophil CD64 (nCD64) expression has been found to be a better diagnostic marker for sepsis than procalcitonin (PCT) [6] and C-reactive protein (CRP) [7] in adults and recently in children [8]. An increase in the expression of integrins of the beta 2 subfamily on neutrophils, in particular CD11b, is also considered to be a good marker of cell activation [9]. Contradictory results regarding neutrophil CD11b expression in premature infants [10] and adult patients with sepsis [11] and septic shock [9] have been reported.

Critical illness constitutes a serious derangement of metabolism, hallmarked by hypometabolism [12], protein breakdown, hyperglycemia, and an altered serum lipid profile [13]. It has recently been shown that plasma levels of lipid peroxidation biomarkers increased significantly in the first 70 hours after severe traumatic brain injury (TBI) [14]. Reductions in mitochondrial energetics and brain energy stores resulting from TBI may put extreme stress on brain metabolism to maintain these critical gradients [15]. Systemic inflammation and sepsis are also accompanied by severe metabolic alterations, including insulin resistance together with increased levels of triglycerides (TG) and decreases in high- (HDL) and low-density-lipoproteins (LDL) [16]. Total cholesterol (TC), LDL, HDL, and apolipoproteins were all found to be significantly lower in non-survivors than in survivors adult patients after sepsis criteria had been met for the first time [17]. Low TC and lipoprotein concentrations found in critically ill surgical patients correlated with interleukins 6 and 10, soluble interleukin-2 receptor, and predicted clinical outcome [18]. In vitro as well as in vivo, the addition of lipoproteins almost abrogated the release of proinflammatory cytokines [19,20]. We have also shown that early pharmaco-nutrition might modulate cytokines in children with septic shock [21] or TBI [22], although it could not offer clinical advantage over the one demonstrated by non-immune enhancing enteral nutrition. In addition, the cytokine response in SIRS or sepsis was reliably reflected by increases in prognostic inflammatory and nutritional indices but not by the metabolic patterns [12]. CRP, however, was shown to promote atherogenesis through inducing lectin-like oxidized low-density lipoprotein receptor-1 an endothelial receptor that plays a pivotal role in endothelial dysfunction [23].

Cellular membranes contain microdomains known as ‘lipid rafts’ or detergent-insoluble microdomains enriched in cholesterol and sphingolipids. Among other T cell or B cell receptors, CD64 have been shown to reside within lipid rafts without prior triggering of the receptor [24]. CD64 was also shown to co-patch with GM1, a microdomain-enriched glycolipid, and that depletion of cellular cholesterol, modulated CD64-ligand interactions [24]. Increased detergent resistant fraction of membranes-association of the Fc gamma RI receptor CD64 and the complement receptor 3 complex CD11b/CD18 were observed from patients with SIRS/sepsis or coronary artery disease myocardial infarction [25]. Flow cytometry studies using CD16, CD32 and CD64 monoclonal antibodies showed a sharp reduction on the expression of CD64 both by human monocyte-derived macrophages and THP-1 cells after incubation with LDL, suggesting preferential engagement of this type of Fc receptor [26]. Assessment of early (first 6 hours) metabolic patterns in critically ill children with different sepsis severity or severe TBI and their relation to the main white blood cells (WBC) surface CD64 and CD11b expressions has not been previously attempted. Presumed association of sepsis and/or SIRS with an acute “metabolic syndrome” might open new horizons in the research of early metabolic disturbances in acute stress states, aiming at studying combined immunological and nutritional interventions in sepsis and other critical illnesses.

This study tests the hypothesis that early stress-metabolic patterns in children with severe sepsis or TBI (non-infectious SIRS) differ, compared to the non-stressed metabolism of healthy children. The study also aims to elucidate whether the acute stress (first 6 hours) plasma concentrations of glucose, TG, TC, HDL or LDL are associated with increased CD64 and/or CD11b expression on neutrophils, monocytes, and lymphocytes in children with sepsis (S) and severe sepsis/septic shock (SS) as compared to those with severe TBI or healthy controls (C).

## Methods

### Patients and setting

This prospective observational study was conducted in the University Pediatric Intensive Care Unit (PICU) between January 2010 and June 2011. Forty-five critically ill children admitted to the PICU with clinical diagnosis of TBI associated SIRS (n = 15), S (n = 15), and SS (n = 15) were enrolled in the study within 6 hours of admission. Fifteen healthy children undergoing screening tests for minor elective surgery were used as the control group. Allocation into groups was carried out by the, unaware of the results of flow cytometry, attending physician until 15 children were included in each group. Although only 3 of the severe sepsis group presented with septic shock, all severe sepsis and septic shock patients had SIRS, infection and more the 2 organ system failures, thereby representing the homogenous SS group. Our trauma patients were TBI with severe head injury and almost all had additional minor non-life threatening injuries (lung contusions, fractures, liver or spleen lacerations). Patients’ characteristics and outcome endpoints, as are the length of stay (LOS) and length of mechanical ventilation (LOMV), are summarized in Table 1. Patients were on standard glucose solutions with electrolytes during the first 6–12 hours upon admission. After initial blood studies, all patients were put on isocaloric - isonitrogenous enteral nutrition. None of the patients received parenteral nutrition. The Pediatric Risk of Mortality Score (PRISM) [27], the Therapeutic Intervention Scoring System (TISS) modified for children [28], and the Pediatric Logistic Organ Dysfunction Score (PELOD) were calculated as markers of severity of disease on the day of blood sampling [29]. Sepsis, severe sepsis, septic shock, and SIRS due to traumatic brain injury were defined according to the International Pediatric Sepsis Consensus Conference definitions [30]. Thirty-seven patients with malignancies, metabolic or endocrine diseases (diabetes, hypercholesterolemia) or immune-compromised (immunosuppressive agents but not stress-dose hydrocortisone) were excluded.

**Table 1:** Characteristics of the study population

	**Control**	**TBI**	**Sepsis**	**Severe sepsis**	**p value**
**Number**	15	15	15	15	
**Gender Male/Female**	11/4	9/6	8/7	9/6	0.3
**Age [years]**	8 (2–14)	7.5 (2–15)	3.75 (1–18)	7 (1–14)	0.26
**Mortality**	0	2	0	0	0.09
**PRISM II**	NA	9.5 (6–29)	11 (2–20)	16 (6–26)	0.06
**PELOD**	NA	2.5 (1–21) ^b^	2 (1–13) ^c^	14 (10–32) ^b, c^	0.006 ^a^
**TISS**	NA	41 (26–57)	36.5 (25–47)	44 (32–49)	0.3
**LOS [days]**	NA	7 (3–18) ^b^	10.5 (3–98)	21 (6–235) ^b^	0.02 ^a^
**LOMV [days]**	NA	5 (2–14) ^b^	6 (0–95)	15 (0–235) ^b^	0.13

Results are given as Median (range).

^*a*^*Statistically significant differences, Kruskal-Wallis test.*

^*b, c*^*Post-hoc Mann–Whitney U-test between 2 groups (p < 0.03).*

TBI, Traumatic Brain Injury; PRISM, Pediatric Risk of Mortality; TISS, the Therapeutic Intervention Scoring System; PELOD, Paediatric Logistic Organ Dysfunction; LOS, Length Of Stay; LOMV, Length Of Mechanical Ventilation.

Admission blood and urine cultures, bronchoalveolar lavage (BAL), and pharyngeal and deep tissue swabs were taken in all critically ill patients, although most of our patients had already been on antibiotics prior to their admission to the PICU. All traumatic brain injury patients had severe head injury (Glasgow Coma Scale (GCS) between 5 and 9, with continuous intracranial pressure monitoring).

### CD64 and CD11b expression assays-flow cytometry

Blood was drawn for routine exams at the time of admission. Venous blood was processed to measure WBC, PCT, and CRP levels. Whole blood EDTA-anticoagulated samples were immediately transported to the laboratory for flow cytometric CD11b and CD64 analysis. More specifically 100 μl of the whole blood sample were incubated with both fluoroscein isothiocyanate (FITC) - conjugated CD11b (Clone Bear1) and R- Phycoerythrin (PE) - conjugated CD64 (Clone 22) for 20 min, in the dark, at room temperature. FITC and PE –mouse IgG was used as isotype control (antibodies from IMMUNOTECH- *BECKMAN COULTER*, Marseille, France). Labeled cells were thoroughly washed with PBS + 2% FCS + 0.05% Sodium Azide. Following lysis in Immunoprep Reagent System (BECKMAN COULTER, Mervue, Galway, Ireland) they were analyzed on an Epics-Coulter cytometer. Cells were initially gated according to their morphology (side- and forward-scatter characteristics) and more than 30000 cells were acquired in each sample. Results are presented as the percentage (%) of positive-staining cells. Analysis was performed in lymphocytes, monocytes and polymorphonuclear cells.

### Routine laboratory measurements

CRP assays were performed on the Dade Behring BN II immunonephelometer (Dade Behring Diagnostics Inc., Somerville, NJ). CRP levels >0.8 mg/dL were considered abnormal. PCT was measured by an immunoluminometric assay (Lumitest-PCT, Brahms-Diagnostica, Berlin, Germany). Detection threshold was 0.1 ng/mL; PCT levels >0.5 ng/mL were considered abnormal (Laboratory cut off values). Plasma concentrations of glucose, TG, TC, HDL or LDL, and hematological markers were measured by routine laboratory methods. All nutritional indices and acute phase proteins were repeated on day 2 and 3 after admission in S, SS and TBI groups (n = 150 including day 1 group C).

### Research ethics

The study was approved by the Ethical Committee of the University Hospital, Heraklion. Written informed consent was obtained from the parents before inclusion in this study. All data were anonymous.

### Statistical analysis

The one-sample Kolmogorov–Smirnoff test was used to determine the distribution of data from measured parameters. Data are presented as the median and range. Group comparisons were performed by using the Kruskal-Wallis test. The variables that showed differences among the 4 groups were compared group-by-group applying the Mann–Whitney test. Friedman’s two-way analysis of variance by ranks for non-parametric related-samples was used for comparing the 3-day-series measurements. The correlation between variables was analyzed by the Spearman correlation test. Proportions of patients were compared by the *χ*^2^ test. A two-sided alpha of 0.05 was used for statistical significance. The results were analyzed using SPSS software (version 20.0, SPSS, Chicago, Ill).

## Results

There were no statistical differences regarding gender and age in the studied groups. Only 2 patients in the TBI group died, both having suffered multiple injuries from motor vehicle accidents (patients with the highest PRISM and TISS). Patients with severe sepsis had higher severity scores and longer stay in PICU or duration of mechanical ventilation compared with the sepsis and trauma groups (Table 1).

In the two septic groups, pathogens isolated were as follows: blood: *Streptococcus pneumoniae (n = 2), Staphylococcus haemolyticus (n = 2), Neisseria meningitidis (n = 1), Acinetobacter baumannii (n = 2), and Candida species (n = 1); BAL: Pseudomonas aeruginosa (n = 4), Serratia marcescens (n = 1), Stenotrophomonas maltophilia (n = 2), and Candida species (n = 5); urine: Pseudomonas aeruginosa (n = 2);* central nervous system: *Neisseria meningitidis (n = 1)*. Patients without isolates had received antibiotics before PICU admission. No pathogens were isolated from the blood and samples from other sites in TBI patients.

**Figure 1:**
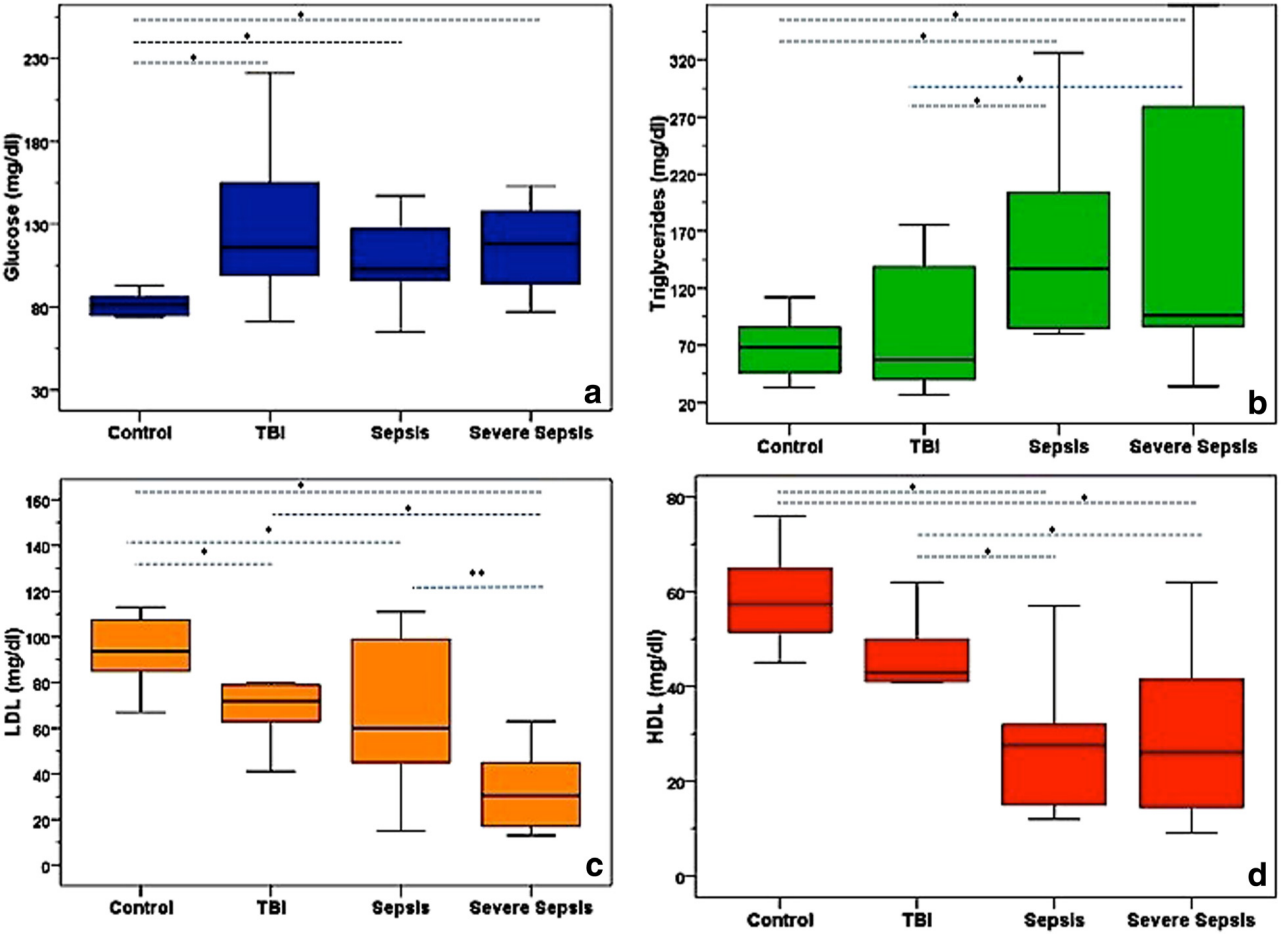
**Early phase metabolic patterns were characteristic of the pathologic process significantly differing between sepsis and TBI. **Box plots of **a**) glucose (GL), **b**) triglycerides (TG), **c**) low-density-lipoproteins (LDL), and **d**) high-density-lipoproteins (HDL) in healthy children and in patients with systemic inflammatory response syndrome (SIRS) after Traumatic Brain Injury (TBI), sepsis (S), or severe sepsis (SS). Metabolic patterns differed among the four groups: TG levels were significantly increased and TC, LDL, and HDL significantly lower in septic compared to C and/or TBI groups (p < 0.01)*. GL did not differ between TBI and sepsis groups, whereas only LDL differed between S and SS (p < 0.02)**. The bold black line in box plots indicates the median per group, the bottom of the box indicates the 25th percentile and the top of the box represents the 75th percentile; the T-bars (whiskers) and horizontal lines show minimum and maximum values of the calculated non-outlier values.

WBCs and platelets did not differ among groups. Although glucose levels were not different among septic and TBI groups, patients of each of the 3 critically ill groups had significantly increased glucose levels compared to controls. Admission levels of CRP, PCT, TG and the TC/HDL ratio were significantly increased in S and SS compared to TBI and C groups (Table 2). Significantly lower were the levels of TC, LDL, and HDL in septic groups compared to C and even to TBI (moderately decreased levels). However, only LDL could discriminate between the two septic groups (p < 0.02) (Figure 1). Although 2 patients with candidemia had low PCT and/or CRP levels (outliers), despite high CD64 expression, differences between bacterial and candida septic patients did not reach statistical significance in any of the measured indices.

**Table 2:** Admission acute phase proteins, platelets, and metabolic indices in children with SIRS compared to healthy controls

	**Control**	**TBI**	**Sepsis**	**Severe sepsis**	**p value**
	Values: medians (ranges)				
**CRP (mg/dl)**	0.3 (0.1-0.7) ^b,c,d^	0.3 (0.3-11) ^c,e,f^	6.4 (0.5-15) ^d,e^	12.8 (3.1-41) ^b,f^	0.0001^a^
**PCT (ng/ml)**	0.2 (0.1-0.4) ^b,c,d^	0.5 (0.1-2) ^c,e,f^	8 (0.3-12) ^d,e^	11.5 (0.3-12) ^b,f^	0.0001^a^
**WBC**	9.7 (8.4-11)	12.3 (4.4-28)	20.8 (2–43)	9.9 (1.5-47)	0.31
**Platelets (K/μl)**	256 (212–300)	288 (135–518)	285 (105–801)	215 (24–451)	0.33
**Glucose (mg/dl)**	83 (74–108) ^b,c,d^	134 (71–243) ^c^	115 (65–202) ^d^	118 (77–153) ^b^	0.002^a^
**TG (mg/dl)**	71.8 (33–150) ^b,d^	79.2 (27–175) ^e,f^	151.7 (80–326) ^d,e^	209.1 (34–591) ^b,f^	0.007^a^
**TC (mg/dl)**	171.3 (133–235) ^b,c,d^	132 (98–166) ^c, f^	125.2 (56–208) ^d^	100.6 (45–150) ^b, f^	0.0001^a^
**LDL (mg/dl)**	98.1 (57–155) ^b,c,d^	72.2 (41–110) ^c f^	66.2 (15–111) ^d,g^	33.4 (13–63) ^b,f,g^	0.0001^a^
**HDL (mg/dl)**	58.7 (45–76) ^b,c,d^	45.3 (24–62) ^c,e f^	27.3 (12–57) ^d,e^	29.5 9–62) ^b,f^	0.0001^a^
**TC/HDL (ratio)**	2.9 (2.3-3.8) ^b,d^	3.1 (2.1-5.2) ^c f^	5.0 (3–8.7) ^d,c^	5.5 (2.67-12.1) ^b,f^	0.05^a^

Results are given as Median (range).

^*a*^*Statistically significant differences, Kruskal-Wallis test.*

^*b –g*^*Post-hoc Mann–Whitney U-test between 2 groups (p < 0.05).*

SIRS, systemic inflammatory response syndrome; TBI, Traumatic Brain Injury; CRP, C-Reacting Protein; PCT, Procalcitonin; TG, Triglycerides; TC, Total Cholesterol; LDL, Low-Density-Lipoproteins; HDL-Density-Lipoproteins; WBC, White Blood Cells.

**Figure 2:**
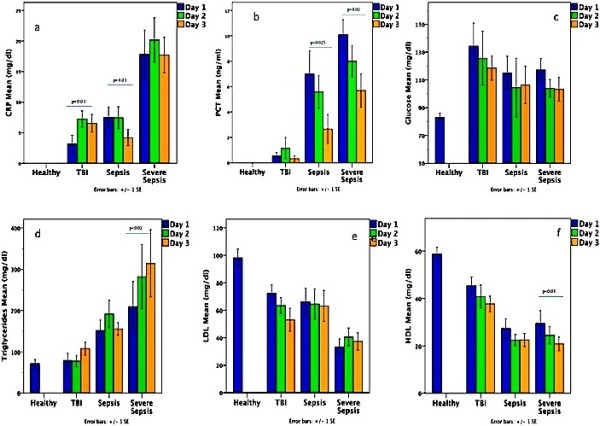
**Early lipid disturbances persisted for3-days in both, the septic and the traumatic metabolic pattern. **First 3 days distribution of **a**) C-reactive protein (CRP), **b**) procalcitonin (PCT), **c**) glucose (GL), **d**) triglycerides (TG), **e**) low- (LDL) and **f**) high-density-lipoproteins (HDL) in healthy children and in patients with systemic inflammatory response syndrome (SIRS) after Traumatic Brain Injury (TBI), sepsis (S), or severe sepsis (SS). The 3-day follow up showed a significant longitudinal decrease of PCT in septic patients followed by parallel decreases of HDL and increases of TG in the SS group. CRP changes in TBI and S groups were not reflected by similar changes in any of the metabolic indices (differences shown by horizontal lines). Pathologic metabolic patterns were distinct in the sepsis and TBI groups compared to controls and all early (first 6-hours) HDL and TG differences between S or SS and TBI persisted throughout the first 3 days (p < 0.04); LDL day 2 was lower in SS compared to TBI (p < 0.03). All 3 days PCT and CRP differed between TBI and SS (p < 0.005); PCT, but not CRP, differed between TBI and S on day 2; in contrast, only PCR differed between S and SS on days 2 and 3 (p <0. 001).

Glucose, TC, and LDL levels did not differ among days 1, 2 or 3 in any of the SIRS groups (Figure 2). Among septic patients, first 3 days PCT levels declined significantly (p < 0.02), followed by parallel decreases of HDL (p < 0.03) and increases of TG (p < 0.02) in the SS group. CRP changes in TBI and S groups were not reflected by similar changes in any of the metabolic indices (Figure 2). All 3 days PCT and CRP differed between TBI and SS (p < 0.005) but only PCR differed between S and SS on days 2 and 3 (p <0. 001). All 3 days HDL and TG differed between S or SS and TBI (p < 0.04); LDL day 2 was lower in SS compared to TBI (p < 0.03).

There was no difference in the neutrophil and monocyte CD11b expression among groups in all 4 groups studied. Lymphocyte CD11b showed a trend for lower values in SS compared to C and TBI or S (Table 3). Admission monocyte CD64 did not vary among groups. Lymphocyte CD64 was found extremely low as expected. Although admission neutrophil CD64 expression did not differ between the two septic groups it was significantly higher in patients with SS or S as compared to TBI and C (Table 3).

**Table 3:** Admission CD11b and CD64 (%) for neutrophils, monocytes, and lymphocytes in 3 groups of critically ill children compared to healthy controls

	**Control**	**TBI**	**Sepsis**	**Severe sepsis**	**p value**
	Values are medians (ranges)				
**Neutrophils**					
***CD11b (%)***	99.6 (98–100)	98.9 (93–100)	99 (92.6-99.9)	99.6 (96.3-100)	0.29
***CD64 (%)***	0.9 (0.1-4.3) ^b,c,d^	5.5 (3–11.3) ^c,e,f^	78.8 (25.7-97.3) ^b,e^	81.2 (17.5-97.8) ^d,f^	0.0001^a^
**Monocytes**					
***CD11b (%)***	96 (86–99)	96.4 (92–99)	95 (52–99)	87.4 (24–97)	0.11
***CD64 (%)***	87.4 (78–94)	87.9 (87–97)	90.9 (42–99)	87.3 (18–94)	0.52
**Lymphocytes**					
***CD11b (%)***	22.9 (7.7-36.5)	18.3 (8.2-39.5)	16.8 (7.3-42)	9.3 (0.9-33.5)	0.043^a^
***CD64 (%)***	0.1 (0–2.6) ^b,c^	0.6 (0.1-3.8) ^c^	0.82 (0.3-1.5) ^b^	0.25 (0.1-2.9)	0.01^a^

Results are given as Median (range).

^*a*^*Statistically significant differences, Kruskal-Wallis test.*

^*b –f*^*Post-hoc Mann–Whitney U-test between 2 groups (p < 0.03).*

TBI, Traumatic Brain Injury.

**Figure 3:**
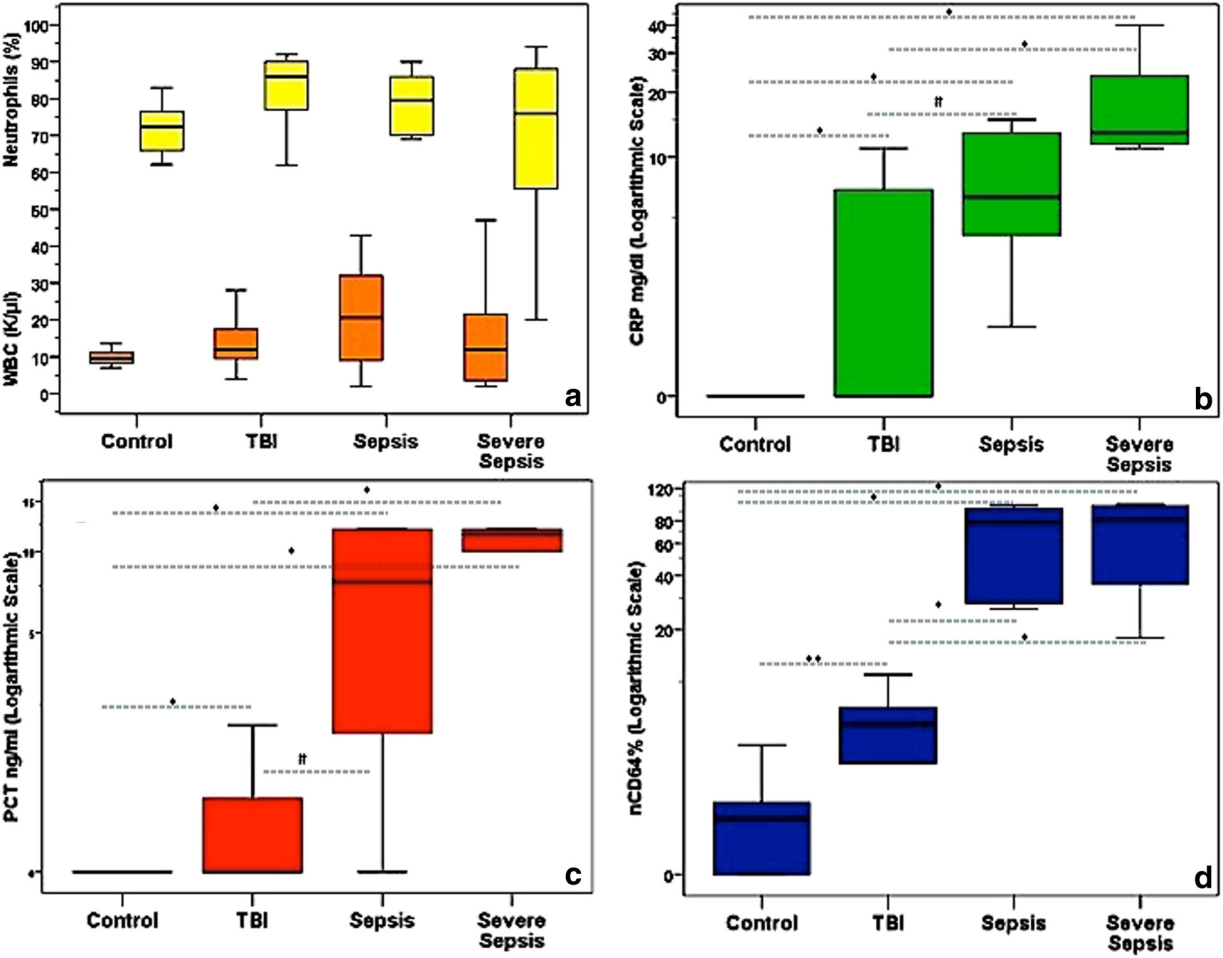
**Neutrophil CD64 expression responded to acute stress not to white blood cell or neutrophil activation. **Box plots of **a**) White blood cells (WBC) and Neutrophils, **b**) C-reactive protein (CRP), **c**) procalcitonin (PCT), and **d**) Fc gamma receptor expression on neutrophils (nCD64) in healthy children and in patients with systemic inflammatory response syndrome (SIRS) after Traumatic Brain Injury (TBI), sepsis (S), or severe sepsis (SS). CRP and PCT differed between the septic groups and C (p < 0.001)* and/or TBI groups (p < 0.03)^#^. In contrast to the WBC and neutrophils, which did not differ among groups, nCD64 differed between septic groups and C or TBI groups (p < 0.001)* and between TBI and C groups (p < 0.02)**. The bold black line in box plots indicates the median per group, the bottom of the box indicates the 25th percentile and the top of the box represents the 75th percentile; the TBI-bars (whiskers) and horizontal lines show minimum and maximum values of the calculated non-outlier values.

Neutrophil CD64 expression differences were not influenced by WBC or neutrophil population variations among groups, rather paralleling a similar incremental stress-response of acute phase proteins (Figure 3). Neutrophil CD64 was positively related with serum levels of CRP (r_s_ = 0.82 p < 0.0001), PCT (r_s_ = 0.89, p < 0.0001), glucose (r_s_ = 0.42, p < 0.01), and TG (r_s_ = 0.46, p < 0.008) and negatively with TC (r_s_ = −0.60, p < 0.0001), LDL (r_s_ = −0.62, p < 0.0001), and HDL (r_s_ = −0.70, p < 0.0001). CD64 did not correlate with any of the severity of illness scoring systems, GCS, hematologic indices, LOS or LOMV. No correlation was shown between neutrophil CD11b and any of the severity of illness, acute phase, and metabolic or hematologic indices.

## Discussion

We showed that the nCD64 expression associated with an acute phase metabolic response pattern in severe TBI is different from the one of early sepsis, whose rapid disturbance is evident upon admission. The high^glucose – TG^ – low_TC - LDL –HDL_ stress metabolic pattern of early septic groups differs from the moderate ^glucose^–moderate _TC - LDL – HDL_ stress pattern of severe TBI-related SIRS. We showed that metabolism is disturbed early (within 6 hours) and differently in SIRS groups (infectious and non-infectious) compared to the normal metabolic pattern of healthy individuals. We also showed that early metabolic patterns persist during of acute illness, showing the same first 3 days TG, HDL and first 2 days LDL differences among groups. In addition, acute phase PCT, but not CRP levels, decline steadily in the SS group followed by parallel decrease of HDL and increase of TG. The decreases in PCT levels but not in CRP levels during the three days is probably due to the different kinetics of both biomarkers. However, neutrophil only, but not lymphocyte or monocyte, CD64 expression is higher in septic children as compared to those with TBI or C. Finally we showed that admission CD11b expression on neutrophils and monocytes is not diagnostic and non-related to the metabolic patterns, although its expression on lymphocytes may be suppressed in the presence of severe sepsis.

These results are comparable to those of previous studies in adults [4,31], children [8], or neonates [32], which have shown that nCD64 expression was higher in patients with bacterial SIRS compared to non-bacterial SIRS [33]. It is the first time, though, that all 3 cell lines’ CD64 expression were simultaneously examined in children with TBI and related to acute phase metabolic derangements. Although nCD64 expression has been thought to increase with sepsis severity [34], we did not find nCD64 expression to be influenced by severity of illness or even sepsis staging, but by the presence of “sepsis” itself. In our TBI group, CD64 and CD11b were not related to GCS or any of the severity scoring, acute phase, or hematologic indices. We did not find any relation between CD64 and CD11b in all four groups studied; however, during sepsis, cell surface antigen functionality may be expressed independently. This is further supported by recent work showing that CD64 was upregulated, CD16 downregulated, and CD32 remained unchanged during sepsis [35]. In support to our results, no differences were found in CD11b expression on neutrophils and monocytes among adult healthy volunteers, patients with sepsis, severe sepsis and septic shock [36]. It is not therefore surprising, that none of the 3 cell lines expressing CD11b in our series could differentiate critically children in stress from healthy controls. Nonetheless, if it has been technically possible to follow CD64 and CD11b levels during the first admission days, their evolution could have probably offered higher accuracy.

Although the CD64 index was shown to be specific for bacterial infection among ICU patients, its weak sensitivity demanded its combination with a more sensitive biological marker [37]. This is the first time that the CD11b integrins and the CD64 high-affinity receptors expressed on lymphocytes, monocytes, and neutrophils are related to metabolic patterns in critically ill patients. In contrast to the critically ill adult patients, in which no significant differences in TG were found between infectious and non-infectious disease patients [38], we showed an early glucose-TG increase in both sepsis groups in contrast to the hyperglycemia only TBI pattern. These different metabolic patterns were characterized by an early significant decrease of TC, HDL, LDL in the septic groups compared to moderate cholesterol - lipoprotein decrease of TBI and the normal levels of C groups. In an observation study in adults, low HDL also pointed to bacterial infection and low TC was predictive of adverse outcomes in patients with lower respiratory tract infections [39]. Our results confirm findings of a recent adult study, showing that the levels of TC, LDL, and HDL were highly significantly lower than in controls, especially in the most severely affected septic groups [40]. Although rapid decline was primarily described in the HDL, suggesting a decline in lipoprotein particles [41], we found a rapid (within 6 hours) decline in both HDL and LDL fractions and also in TC accompanied by early increase in glucose, TG associated with increased nCD64 expression. We showed for the first time that the TC/HDL ratio, a robust measure of cardiovascular disease risk [42,43], is steadily increased in the context of acute stress in children with sepsis or severe TBI. We also showed that the steadily declining acute phase PCT levels in the SS group were followed by parallel decreases of HDL and increases of TG; importantly, only LDL differed between S and SS groups. The clinical importance of these results however should be further evaluated in larger multicenter studies.

A strong positive relation between nCD64 and TG or glucose and a negative one with TC, HDL, and LDL may suggest a direct rapid effect of the inflammatory process on metabolism. It is possible that triglycerides rapidly respond in sepsis in an effort to protect from endotoxin, since lipoprotein- lipopolysaccharide (LPS) binding is associated with an attenuated response to this toxic macromolecule [44]. Approximately 80% of the LPS was bound to lipoproteins after preincubation with either fasting or hypertriglyceridemic blood; in addition, hypertriglyceridemic but not fasting blood inhibited the ex vivo TNF-alpha response to large, highly toxic doses of LPS [44]. Similarly, a multivariate logistic regression analysis, corrected for baseline univariate risk factors and the effect on inflammation, indicated that lipid rather than glucose control independently determined the beneficial effects of intensive insulin therapy on morbidity and mortality [45]. The different metabolic patterns we noted also point to the importance of TG metabolism rather (high in S and SS only) than the one of glucose (similar in all SIRS groups). Thus, although early hyperglycemia in TBI has been linked to increased catecholamine concentrations and lower GCS score [46], we did not find any association of admission glucose levels to the severity scoring systems or acute phase proteins. However, our finding of low TC not only in sepsis but also in TBI was further supported by results of a recent study showing substantial hypocholesterolemia in TBI greater with shock but less with brain injury [47].

It has been recently reported that 62% of hyperglycemic septic children had overt insulin resistance on admission and 17% had β-cell dysfunction [48] and that the endotoxin activity was significantly associated with 52% increased risk for incident diabetes [49]. Further studies are needed to examine the cross talk between hormonal and inflammatory responses in the development of SIRS in sepsis, since the metabolic and immune systems are closely involved in inflammation and may significantly contribute to the critically illness pathology [1]. Although limited by the small sample size, our study might contribute in setting up the stage for larger cohort of patients; the precise role of these molecules and their possible implications in the manipulation of the metabolic disturbances should be further examined and provide new data for the management of sepsis and possible nutritional and therapeutic targets for the critically ill.

## Conclusions

Acute-phase stress-metabolic pattern in traumatic brain injury, thereof a non-infectious SIRS, is different from the one of sepsis, whose response is evident within 6 hours of admission associated with increased acute phase proteins. The early sepsis stress-metabolic pattern is characterized by a high (nCD64, glucose, TG) - low (TC, HDL, LDL) combination in contrast to the TBI in which with the exception of an increased glucose, lipoprotein decrease is moderate and nCD64 or TG increases are not evident. Importantly, early metabolic patterns persist during the first 3 days of acute phase of sepsis or TBI. Early neutrophil, but not lymphocyte or monocyte, CD64 expression differentiates children with sepsis from those with trauma and is associated with high CRP, PCT.

### Key messages

• Metabolism is early and differently disturbed in infectious and non-infectious SIRS in critically ill children

• In sepsis, the early stress-metabolic pattern is characterized by a high (nCD64, glucose, TG) - low (TC, HDL, LDL) combination

• TBI early stress-metabolic pattern is moderate, combining glucose increase with moderate cholesterol - lipoprotein decrease

• Early stress-metabolic patterns are associated with the acute phase CD64 expression on neutrophils, but not on lymphocytes or monocytes or with the CD11b expression

• Early acute phase stress-metabolic patterns of sepsis and TBI persist the first 3 days of acute illness

## Abbreviations

TBI: Traumatic brain injury; S: Sepsis; SS: Severe sepsis/Septic shock; C: Healthy controls; CRP: C-Reactive protein; PCT: Procalcitonin; SIRS: Systemic inflammatory response syndrome; TG: Triglycerides; TC: Total cholesterol; HDL: High-density-lipoproteins; LDL: Low-density-lipoproteins; nCD64: Neutrophil CD64; WBC: White blood cells; PICU: Pediatric intensive care unit; LOS: Length of stay; LOMV: Length of mechanical ventilation; PRISM: Pediatric risk of mortality score; TISS: Therapeutic intervention scoring system; PELOD: Pediatric logistic organ dysfunction score; BAL: Bronchoalveolar lavage; GCS: Glasgow coma scale.

## Competing interests

The authors declare that they have no competing interests.

## Authors’ contributions

GB and DMF contributed to the study conception and design, recruited the patients, carried out and participated in data analysis and drafted the manuscript. HD carried out flow cytometry assays and managed the data, including quality control. HD and MK reviewed and edited the manuscript and GB supervised the conduct of the trial and the data collection and reviewed and edited all versions of the manuscript. All authors contributed substantially to its revision and approved the final manuscript.

## Pre-publication history

The pre-publication history for this paper can be accessed here:

http://www.biomedcentral.com/1471-2431/13/31/prepub
